# Tokyo Guidelines (TG18) for Acute Cholangitis Provide Improved Specificity and Accuracy Compared to Fellow Assessment

**DOI:** 10.7759/cureus.27527

**Published:** 2022-07-31

**Authors:** Amit Hudgi, Anabel L Cartelle, Amr Ahmed, Ahmad Alkaddour, Carlos Palacio, Kenneth J Vega, John Erikson L Yap

**Affiliations:** 1 Internal Medicine, Medical College of Georgia - Augusta University, Augusta, USA; 2 Gastroenterology and Hepatology, Medical College of Georgia - Augusta University, Augusta, USA; 3 Internal Medicine, University of Florida, Jacksonville, USA

**Keywords:** tokyo guidelines (tg18), endoscopic retrograde cholangiopancreatography (ercp), in-training fellows, choledocholithiasis, acute cholangitis

## Abstract

Background

Acute cholangitis results in significant mortality unless treated promptly. The diagnostic grading criteria of the 2018 Tokyo Guidelines (TG18) are used worldwide as the standard for acute cholangitis (AC) management but validation in clinical practice is required.

Aim

Use of the Tokyo 2018 (TG18) guidelines in improving the diagnostic accuracy and early detection of AC compared to fellow clinical assessment.

Methods

A retrospective review of patient records from 1/2010-9/2019 seen at Augusta University - Medical College of Georgia with the International Classification of Diseases, Ninth Revision (ICD-9) code “cholangitis” and/or ICD-10 codes “acute cholangitis, other cholangitis, and calculus of bile duct with cholangitis” was performed. Inclusion criteria were gastroenterology inpatient consult fellow evaluation and clinical diagnosis of AC. A definitive diagnosis of AC was determined following endoscopic retrograde cholangiopancreatography (ERCP). TG18 scoring for AC was then performed, categorized as either diagnostic/non-diagnostic, and compared to fellow clinical assessments following definitive diagnosis post-ERCP. Data were analyzed with chi-square testing.

Results

Two hundred six patients were identified using ICD codes. Ninety-one met inclusion criteria and were analyzed. The mean patient age of the overall group was 67 years old (standard deviation of 13.3 years) with males comprising 69% and non-Hispanic white 56% of the study group. TG18 criteria assessment had a sensitivity of 86% and specificity of 63% for patients with AC post ERCP (p <0.05). TG18 accuracy was 81%. In comparison, fellow clinical suspicion had a sensitivity of 90.3% and specificity of 0% (NS). Fellow accuracy was 71%. No difference in fellows’ diagnosis of suspected AC was noted based on the training year.

Conclusion

Application of the TG18 criteria for AC reduces the false positive rate and improves diagnostic accuracy, thus decreasing costs along with avoiding unnecessary ERCPs with associated complications.

## Introduction

Acute cholangitis (AC), also known as ascending cholangitis or acute ascending cholangitis, is a serious medical condition characterized by inflammation and infection of the bile duct system [1]. In the United States, AC affects males and females equally and, on average, presents most frequently in patients in the 50 to 60-year age range. It remains a relatively uncommon condition with fewer than 200,000 cases reported annually [2]. Failure to detect AC early in its disease course can be life-threatening. Thus, the ability of providers to promptly diagnose AC is of vital importance. Despite Charcot having described AC more than 100 years ago, there remains no gold standard method for AC diagnosis [3]. Traditionally, AC has been diagnosed clinically using Charcot’s observations of a symptom triad consisting of fever, jaundice, and right upper quadrant (RUQ) abdominal pain. More severe cases were further characterized by the presence of hypotension and altered mental status, completing Reynolds’ pentad [4].

Unfortunately, clinical presentations of Charcot’s triad and Reynold’s pentad have limited utility in the diagnosis of AC. For Charcot’s triad, a systematic review yielded a sensitivity of 36.3% derived from 16 studies, and a specificity of 93.2% gathered from three studies [5]. Reynolds’ pentad proved to be even more inferior with a reported sensitivity of 4.82% and unknown specificity [5]. Improved understanding of hepatobiliary physiology and a corresponding elucidation of AC pathophysiology has led to a revaluation of salient diagnostic criteria. Currently, the development of AC is attributed to prolonged cholestasis secondary to biliary obstruction. The obstruction itself can have varied etiology, ranging from relatively benign choledocholithiasis to malignancy [6]. Regardless, the stagnation of bile in the biliary ductal system promotes the growth of coliform bacteria such as E. coli and Klebsiella [3]. The subsequent increase in intraductal pressures secondary to this stagnation causes a widening of the tight junctions found between cholangiocytes, allowing the transfer of organisms and toxins from the “external” ductal environment to the “internal” milieu [2,7]. Resultant bacteremia and subsequent toxemia then trigger the release of systemic inflammatory mediators leading to hemodynamic instability, septic shock, and ultimately death [1].

At present, the diagnostic grading criteria in the 2018 Tokyo Guidelines (TG18) are considered standard for the management of AC, clinical validation through use in actual clinical practice is still required in regions outside of Asia. The aim of this study was to determine if the usage of Tokyo Guidelines 2018 improves diagnostic accuracy and assists in the earlier detection of AC by a fellow clinical assessment in a single United States academic medical center.

## Materials and methods

Patient selection and data collection

All patients evaluated from January 1, 2010, to September 30, 2019, at Augusta University - Medical College of Georgia was reviewed for the present investigation. Any patient with the International Classification of Diseases, Ninth Revision (ICD-9) code “cholangitis” and/or ICD-10 codes “acute cholangitis, other cholangitis, and calculus of bile duct with cholangitis” were eligible for study inclusion. Inclusion criteria were anyone evaluated by the gastroenterology inpatient consult fellow and given the diagnosis of ACs based on clinical assessment. A definitive diagnosis of AC was determined following endoscopic retrograde cholangiopancreatography (ERCP). TG18 scoring for AC (Table 1) was then performed and categorized as either diagnostic (positive) or non-diagnostic (indeterminate or negative) and compared to fellow clinical assessments following definitive diagnosis post ERCP. Exclusion criteria were patients admitted for surgical evaluation, diagnosed with choledocholithiasis prior to the fellow evaluation, or admitted for biliary catheter exchange. After carefully reviewing the cases, subjects with exclusive choledocholithiasis with no other signs or suspicion of acute cholangitis were excluded from the study. Data collected from the electronic medical record included age, sex, race, presence of fever, right upper quadrant pain, altered mental status, white blood cell count, C-reactive protein, total bilirubin, alkaline phosphatase, aspartate transaminase, alanine transaminase, findings from abdominal imaging, including transabdominal ultrasound, computerized tomography and magnetic resonance imaging (MRI), the timing of and findings from endoscopic retrograde cholangiopancreatography (ERCP) as well as training year of fellow involved. On our review of clinical documentation, we noted that none of the fellows used the Tokyo Guidelines to assess for acute cholangitis.

**Table 1:** Tokyo Guidelines (TG18) diagnostic criteria for acute cholangitis Source: [8]

Tokyo guidelines (TG-18) diagnostic criteria for acute cholangitis
A. Systemic Inflammation
1. Fever and/or shaking chills
2. Laboratory data: Evidence of inflammatory response
B. Cholestasis
1. Jaundice
2. Laboratory data: abnormal liver function tests
C. Imaging
1. Biliary dilatation
2. Evidence of the etiology on imaging (stricture, stone, or stent)
Suspected diagnosis: one item in A + one item in either B or C
Definite diagnosis: one item in A, one item in B, and one item in C

Definitions used

AC was defined as frank pus extruded from the common bile duct (CBD) following cannulation during ERCP. Fellow training level was defined as follows: the first year, 0-11 months of training; second year, 12-23 months of training; and third year, 24-35 months of training. The Charcot’s triad, Reynolds pentad, and TG18 score (Table 1) for AC in each individual case were calculated based on the data above. Time to intervention with ERCP and biliary drainage was divided as follows: <12 hours, 12-24 hours, and > 24 hours after admission.

Statistical analysis and IRB approval

Patients were classified using the TG18 rubric as yes, no, or indeterminate for acute cholangitis. This grouping was dichotomized. One group was categorized as yes. The second group consisted of those who were classified as no or indeterminate. The patients were then assessed as to the clinical diagnosis of cholangitis on discharge from the hospital.

The resulting 2x2 table was analyzed using a chi-square test. A p-value of <5% was considered statistically significant. Sensitivity, specificity, and diagnostic accuracy were calculated from the 2X2 table.

The statistical review was performed by a biomedical statistician. This study was approved by the Institutional Review Board of Augusta University Medical Center, IRB approval number 1559180-2.

## Results

Two hundred six (206) patient records were identified using the ICD-9 or ICD-10 codes listed above. A total of 91 met inclusion criteria and were included in the analysis. Demographic information about the study group is illustrated in Table 2. The mean patient age of the overall group was 67 years old (standard deviation of 13.3 years) with males comprising 69% and non-Hispanic white 56% of the study group. Of the 91 patients, AC was confirmed by ERCP in 72 patients (79%) and seven (7.7%) patients died within 30 days of presentation.

**Table 2:** Baseline characteristics of the patients

Baseline characteristics (N=91)	
Age	67.07 +/- 13.34 years
Male/Female	63 (69.23%)/28 (30.7%)
Race	
Non-Hispanic white	51 (56.04%)
African American	23 (25.27%)
Asian	2 (2.19%)
Other/not reported	15 (16.48%)
30-days mortality	7 (7.69 %)

Based on the application of TG18 criteria before the ERCP procedure, definitive AC was diagnosed in 69 patients, suspected AC was diagnosed in 11, and AC excluded in 11 patients.

Analysis comparing the true diagnosis of the AC with that of the TG18 criteria for definitive diagnosis was statistically significant (p<0.05, Table 3). Results were significant with a sensitivity of 86.1 % and specificity of 63.2%. The diagnostic accuracy was 81.3%. With a presumed prevalence of 79%, the negative predictive value was 63.8% and the positive predictive value was 84.91% (Table 3). The fellows’ diagnosis showed a sensitivity of 90.3% with 0% specificity, which was not significant (p > 0.157). Diagnostic accuracy for fellows was 71.3%. No difference in fellows’ diagnosis of suspected AC was noted based on the training year.

**Table 3:** Analysis of the results with the application of TG18 (definitive diagnosis) vs suspected diagnosis by in-training GI fellows

Statistical Results		
	Definitive TG-18	Fellows’ diagnosis
Sensitivity	86.11%	90.28%
Specificity	63.16%	0.00%
Positive Likelihood Ratio	2.34	0.9
Negative Likelihood Ratio	0.22	
Disease Prevalence	79.00%	79.00%
Positive Predictive Value	89.79%	77.25%
Negative Predictive Value	54.73%	0.00%
Accuracy	81.29%	71.32%
Significance (p-value)	p<0.05	p<0.157

Analysis for the interval to ERCP and biliary drainage revealed intervention of lesser than 12 hours performed in 47% of cases, 34.7% performed within 12-24 hours, and 25% of cases were performed after 24 hours.

Mortality within 30 days was not statistically significantly different among the groups according to the timing of intervention. However, in the group for whom intervention occurred within 12 hours, a trend toward higher mortality was noted in those intervened upon at less than 12 hours (14.3% v. 3.6%, p=.062). This may be a spurious observation or a result of selection bias due to the quasiexperimental design of the study.

## Discussion

Acute cholangitis is a life-threatening condition with mortality ranging from 5% to 30% depending on illness severity and co-morbidities of the patient [9-10]. Considering the dismal diagnostic value of the traditional clinical criteria and improved understanding of AC pathophysiology, experts came together to formulate the Tokyo Guidelines 2007 (TG07), which served as the first diagnostic and severity grading criteria for AC [11]. The criteria improved upon Charcot’s triad by incorporating additional blood tests reflecting inflammation and imaging to confirm biliary obstruction. Subsequent retrospective studies conducted in Japan demonstrated the impact these additional components had on diagnostic sensitivity, increasing from 26.4% using Charcot’s triad to 82.6% with the TG07 criteria [11]. These guidelines were further refined in 2012 to account for shortfalls seen in actual clinical practice for the detection of severe cases and were later validated in a large-scale case series study conducted in Asia to constitute the TG13 criteria. Diagnostic sensitivity improved to 91.8% with the TG13 adjustments [12]. This was such a significant improvement that the most current 2018 iteration of the Tokyo Guidelines did not change the diagnostic criteria for AC [8,11].

Only a handful of studies have effectively analyzed the TG18 criteria for accuracy, and no other studies have addressed its utility by fellows-in-training in an academic center. In the present study, the TG18 criteria were retrospectively applied and compared to fellows’ clinical judgment when consulted with a potential biliary obstruction case. TG18 criteria had higher diagnostic accuracy for AC compared to fellows’ clinical diagnosis. In addition, TG18 guidelines had a higher sensitivity for AC than fellow clinical judgment. However, it is important to acknowledge that the lack of a “gold standard” test limits our ability to obtain true sensitivity to the criteria.

Thus, the incorporation of TG18 would increase accuracy and specificity. Furthermore, TG18 use would improve the identification of true negative cases, avoiding unnecessary ERCP with its potential for complications, including perforation, bleeding, pancreatitis, or rarely AC recurrence, which can range from 6% to 10% [13-14]. In addition, ERCP is an expensive procedure, with one group estimating the economic burden of the procedure per patient between $56,000 and $78,000 [15]. Thus, careful patient selection can lead to lower costs to the health care system as well.

Survival in AC is dependent on timely intervention with biliary drainage. Despite the imperative need to identify this condition, no gold standard test exists. As noted earlier, low sensitivity for AC was seen with both Charcot’s triad and Reynolds pentad, which limits their use in clinical practice. However, an accuracy rate of 92.3% for the TG18 criteria is reported with the recent revision [6]. In addition, sensitivity has also increased incrementally following criteria revisions [3-4,6].

Sub-group analysis of comparing fellows in our academic center based on the training year was insignificant for any difference in diagnosing acute cholangitis. This could be attributed to the “err on the side of caution” approach by in-training gastroenterology fellows. Early intervention has been shown to reduce mortality and hospital stay [16]. In our study, we analyzed the timing of intervention and outcome with in-hospital mortality within 30 days. No difference in mortality was observed based on the timing of intervention from the time of admission.

Limitations of our study include the inherent nature of any retrospective study, the small study sample, and a single-center experience. The absence of confirmatory tests for AC reduces the ability of any investigation to assess the true accuracy. However, the usefulness of this clinical tool was demonstrated for clinical gastroenterology fellows. In addition, to validate this tool for the US population, a large, multicenter, prospective study is required.

## Conclusions

Early diagnosis and treatment are crucial in suspected AC, as untreated AC can be life-threatening. It is also important to weigh in the adverse effects and costs of the invasive ERCP procedure. We determined that utilization of TG18 criteria is a simple and easily accessible tool available for gastroenterology fellows to assist in clinical decision-making in patients potentially with AC. The criteria had higher sensitivity compared to in-training fellows. The application of the tool is readily available and more accurate than the fellows’ assessment in our study.

The use of TG18 criteria can also reduce unnecessary ERCPs, decreasing the risk of complications from unnecessary procedures and health care costs.
